# Human Ovarian Theca-Derived Multipotent Stem Cells Have The Potential to Differentiate into Oocyte-Like Cells *In Vitro*

**DOI:** 10.22074/cellj.2019.5651

**Published:** 2018-08-07

**Authors:** Azam Dalman, Mehdi Totonchi, Mojtaba Rezazadeh Valojerdi

**Affiliations:** 1 Department of Embryology, Reproductive Biomedicine Research Center, Royan Institute for Reproductive Biomedicine, ACECR, Tehran, Iran; 2 Department of Genetics, Reproductive Biomedicine Research Center, Royan Institute for Reproductive Biomedicine, ACECR, Tehran, Iran; 3 Department of Anatomy, Faculty of Medical Science, Tarbiat Modares University, Tehran, Iran

**Keywords:** Differentiation, Mesenchymal Stem Cell, Oocyte, Ovary, Theca

## Abstract

**Objective:**

In this study, we have examined human theca stem cells (hTSCs) *in vitro* differentiation capacity into human oocyte like cells (hOLCs).

**Materials and Methods:**

In this interventional experiment study, hTSCs were isolated from the theca layer of small antral follicles (3-5 mm in size). Isolated hTSCs were expanded and cultured in differentiation medium, containing 5% human follicular fluid, for 50 days. Gene expressions of *PRDM1, PRDM14, VASA, DAZL, OCT4, ZP1, 2, 3 GDF9, SCP3* and *DMC1* were evaluated by quantitative reverse transcription polymerase chain reaction (qRT-PCR) on days 0, 18, and 25 after monoculture as well as one week after co-culture with human granulosa cells (hGCs). In addition, GDF9, OCT4, DAZL, VASA, and ZP3 proteins were immune-localized in oocyte-like structures.

**Results:**

After 16-18 days, the color of the medium became acidic. After 25 days, the cells started to differentiate into round-shaped cells (20-25 µm diameter). One week after co-culturing with hGCs, the size of the round cells increased 60 to70 µm and convert to hOLCs. However, these growing cells expressed some primordial germ cell (PGC)- and germ cell genes (*PRDM1, PRDM14, VASA, DAZL,* and *OCT4*) as well as oocyte specific genes (*ZP1, 2, 3* and *GDF9*), and meiotic-specific markers (SCP3 and DMC1). In addition, GDF9, OCT4, DAZL, VASA, and ZP3 proteins were present in hOLCs.

**Conclusion:**

To sum up, hTSCs have the ability to differentiate into hOLCs. This introduced model paved the way for further *in vitro* studies of the exact mechanisms behind germ cell formation and differentiation. However, the functionality of hOLCs needs further investigation.

## Introduction

The conversion of stem cells into germ cell lineage, probably provides a unique model to identify factors, involved in germ cell formation and differentiation. Consequently, researchers have put a lot of efforts to investigate into the capability of murine/human embryonic stem cells differentiation into primordial germ cells (PGCs) or oocyte-like cells (OLCs) *in vitro* (1, 2).

It has been reported conversion of multipotent stem cells to germ cell-like cells, *in vitro*. These stem cells were originated from the pig or mouse skin, around the time of birth (3), mouse bone marrow mesenchymal stem cells (MSCs) (4), or human adult ovaries (5). Although there is solid evidence for the existence of germ line stem cells in mammalian ovarian tissue; their ability to replenish germ cell pool before or after puberty is still dubious (6). It has been reported that ovarian surface epithelium (OSE) is a possible source of primordial follicle formation during adulthood (7).

White et al. (8), showed that both adult mice and human ovaries possess mitotically active germ cells that are able to differentiate into oocytes both *in vitro* and *in vivo*. Parte et al. (6), 2014 has well recognized the existence of germ cell nests, Balbiani body-like structures and cytoplasmic are indeed well recapitulated during *in vitro* oogenesis in adult OSE cultures. Also, the characteristic expression of stem/germ cell/oocyte markers has been described by them. A subpopulation of mural granulosa cells (GCs) has now been proved to have potential multipotent stem cells (9), which justifies the reason of survival and differentiation of granulosa cells, extracted from preovulatory follicles, over prolonged time of culture periods. In addition to surface epithelium and granulosa cells, studies have proven that theca cells contain multipotent with mesenchymal stem cells-like properties that could differentiate into their lineage cells such as theca cells.

In 2007, Honda et al. (10) purified and differentiated theca stem cells of neonatal mouse ovary *in vitro*. In granulosa cell-conditioned media, these cells show signs of differentiation, lipid droplet accumulation, smooth endoplasmic reticulum formation and production of androstenedione, LH, insulin-like growth factor 1 (IGF1), as well as stem cell factors, later on. Similar to vivo conditions, the transplanted theca cells moved into the mouse ovaries and were surrounded by the growing follicles. It has investigated the cellular properties and the *in vitro* differentiation capacity of porcine ovarian thecaderived multipotent stem cells (11). 

The result of another study revealed that theca stem cells (TSCs) derived from sheep ovarian follicles contain MSCs and pluripotent stem cells (PSCs) that could be differentiated into lineages of mesenchymal origin and are capable of differentiation into theca progenitor cells (TPCs) and OLCs under *in vitro* conditions (12).

We have already isolated and characterized human theca stem cells which are capable of differentiation into human TPCs (hTPCs), *in vitro*. Thus, the aim of present study was to demonstrate the differentiation of hTSCs into hOLCs. This study is the first report of differentiation of hTSCs into hOLCs. 

## Materials and Methods

This study was interventional experiment and the procedures conducted in accordance with the Declaration of Helsinki following approval by the Ethical Committee of Royan Institute (number of ethics committee: EC/93/1059). Patient signed written consent.

## Human theca stem cells isolation and culture 

hTSCs were prepared similar to our previous investigations on sheep (12) and human (13). In brief, small antral follicles, sized 3-5 mm, were collected from the left ovary of a young patient (19 years old) who was referred for ovarian cryopreservation. According to the pathological diagnosis, left ovary, in contrast to the right one, revealed no cancerous cells and was subsequently preserved for further treatment and possible transplantation. Follicles were punctured in Dulbecco’s Modified Eagle’s medium F12 (DMEM/F12, Gibco, Grand Island, NY, USA). 

The oocytes and granulosa cells were removed by scraping. The remaining cells, which were theca layers located in the follicle wall, were dissected into small pieces and incubated with 0.5% collagenase type I (Sigma, USA) for 45 minutes in water bath at 37°C. The enzyme activity was neutralized by adding a medium containing 10% fetal bovine serum (FBS, Gibco, USA). Cell suspension was passed through 100 and 40 µM filters (Millipore) (9). 

Cells were cultured in DMEM/F12 supplemented with 10% fetal bovine serum (FBS), 2 mM glutamine (Gibco, UK), 100 U/mL penicillin G (Sigma, USA), 100 µg/mL streptomycin (Sigma, USA), 20 ng/ml epidermal growth factor (EGF, BioTech, Iran), 10 ng/ml basic fibroblast growth factor (bFGF, BioTech, Iran), 20 ng/ml leukemia inhibitory factor (LIF, BioTech, Iran), and 10 ng/ml glial cell-derived neurotrophic factor (GDNF, BioTech, Iran) at 37°C in a 5% CO_2_ incubator. As soon as hTSCs reached optimal confluence, cells were dissociated in 0.25% trypsin-EDTA (Gibco, USA) and centrifuged at 1200 rpm for 5 minutes and seeded. In order to characterize the hTSCs, at the 3rd passages, alkaline phosphatase assay was performed, cell cycle status and cell surface markers were examined, as well as appearance of osteocyte and adipocyte like cells using induction medium (data not shown here). Finally, with the confirmation of hTSCs, these cells at the 3^rd^ passages were used for further experiments and differentiation into hOLCs. 

## Characterization of human theca stem cells-derived oocyte-like cells

According to the protocol of Dyce et al. (3), hTSCs (7×10^4^ cells) were cultured in DMEM/F12 supplemented with 5% FBS, 5% human follicular fluid, 0.23 mM sodium pyruvate (Sigma, Japan), 0.1 mM non-essential amino acids (Sigma, USA), 2 mM L-glutamine, and 0.1 mM ß-mercaptoethanol (Sigma, USA) for 50 days. This medium was changed twice a week. The morphologically changed round cells were co-cultured with human granulosa cells (hGCs) in oocyte growth medium comprising Minimum Essential Medium Eagle-Alpha Modification (aMEM, Gibco, USA) medium supplemented with 3 mg/mL bovine serum albumin (BSA, MP, France), 1% insulin-transferrin-selenium (ITS, Gibco, USA), 0.005 IU/mL follicle stimulating hormone (Gonal-F, Italy), 0.0025 U/mL human chorionic gonadotropin (Pregnyl, USA), and 0.11 mg/mL sodium pyruvate for 7 days. This medium was changed every other day. For isolation of hGCs, follicular fluids from ICSI-treated patients with male factor infertility were centrifuged at 2000 rpm for 10 minutes. The cell supernatant was removed, and the cells were suspended in up to 2.5 ml of Tyrode salt [0.265 mg/ml CaCl_2_.2H_2_O (Sigma, USA), 0.21 mg/ml MgCl_2_ .6H_2_O (Sigma, USA), 0.2 mg/ml KCL (Sigma, USA), 1 mg/ml NaHCO_3_ (Sigma, USA), 8 mg/ml NaCl (Sigma, USA), 0.05 mg/ml Na_2_HPO_4_ (Sigma, USA), and 1 mg/ml glucose (Sigma, USA) in deionized water], layered slowly onto 2.5 ml Allgrad (LifeGlobal, USA) and 2.5 ml Tyrode salt and then centrifuged at 3000 rpm for 13 minutes. Erythrocytes were precipitated; the interface layer was composed of granulosa cells, lymphocytes, monocytes, and macrophages. The cells were aspirated slowly and then suspended in 5 ml aMEM. Afterward, the pellets were resuspended in 2 ml aMEM with granulosa cell clumps and were treated with 300 µg/ml hyaluronidase for 3 minutes in order to disperse the granulosa cell. Then, the enzyme activity was neutralized by adding medium containing 10% FBS and subsequent centrifugation at 1500 rpm for 7 minutes. The supernatant was removed, and the pellet was resuspended in aMEM containing 10% FBS. Finally, the granulosa cells were cultured in aMEM containing 10% FBS for 4 days and then transferred to oocyte growth medium for the purpose of growing hOLCs. Quantitative reverse transcription polymerase chain reaction (qRT-PCR) and immunostaining were used to analyze the expression of PGC-, germ cell-, oocyte-, and meiotic-related genes at the mRNA and protein levels.

## Gene expression analysis

RNA extraction of hOLCs was performed using an RNeasy micro kit (Qiagen, Germany, Hilden), followed by cDNA synthesis and preamplification using a QuantiTect Whole Transcriptome kit (Qiagen, Germany) according to the manufacture. The expression of *PRDM1, PRDM14, VASA, DAZL, GDF9, OCT4, ZP1, ZP2, ZP3, SCP3,* and *DMC1* was quantitatively measured in hOLCs. *ß-ACTIN* expression was used as a housekeeping gene. The oligonucleotide primers used are listed in Table 1. For each PCR product, the melting curve was determined 2^-ΔΔCt^ using method. The gene expression in the differentiated cells was compared to the undifferentiated cells (controls: day 0) (The number of cells in day 0: 300000 cells, in day 18: 100000 cells, in day 25: 300 cells and seven days after co-culture: 100 cells in each replicate). All qRT-PCR reactions were performed with three biological replicates. 

**Table 1:** Primer sequences and product size for real-time polymerase chain reaction


Gene	Primer sequencing (5ˊ-3ˊ)	Product size (bp)

*DAZL*	F: GTTGTACCTCCGGCTTATTCA	147
	R: GTATGCTTCGGTCCACAGAT	
*DMC1*	F: GACCAATCAAATGACTGCCGA	128
	R: TCTCCTCTTCCCTTTCGCAA	
*GDF9*	F: TAGAAGTCACCTCTACAACACTG	129
	R: GGTAGTAATGCGATCCAGGTTA	
*PRDM1*	F: GCTGACAATGATGAATCTCACA	174
	R: GGGTGAAATGTTAGAACGGTAG	
*PRDM14*	F: AGACTCCCTTCAACTTCCAG	196
	R: GACCATCTTCAAAGATCTCCCA	
*SYCP3*	F: TTACGAGAGCCTATGACTTTGAG	180
	R: ATGTCAACTCCAACTCCTTCC	
*VASA*	F: TGAAATTCTGCGAAACATAGGG	128
	R: TCCCGATCACCATGAATACTTG	
*ZP2*	F: AGCATTGAGGTTGGTGATGG	152
	R: ACTGTTACCTTGCACATAGTGA	
*ZP3*	F: GCCAGATACACTCCAGTTCAC	194
	R: AGATGTCAGCCGAGCCTT	
*ZP1*	F: ACACCTTTCCAGTCGCACTA	101
	R: GCAGAACAAGTAAACCAGTCCT	
*OCT4*	F: CTGGGTTGATCCTCGGACCT	128
	R: CACAGAACTCATACGGCGGG	
*β-ACTIN*	F: CAAGATCATTGCTCCTCCTG	90
	R: ATCCACATCTGCTGGAAGG	


## Immunostaining

hOLCs were fixed with 4% paraformaldehyde solution [diluted in phosphate-buffered saline (PBS)] for 30 minutes, permeabilized with 0.5% Triton X-100 in PBS for 10 minutes at room temperature. Then the cells were incubated with 10% donkey serum in PBS for 30 minutes to block unspecific binding of the antibodies, followed by incubation with the following primary antibodies: OCT4 (mouse monoclonal, Santa Cruz, USA, 1:100), VASA (mouse monoclonal, Abcam, USA, 1:100), DAZL [rabbit polyclonal, Abcam, USA, 1:100 (in blocking buffer)] GDF9 (goat polyclonal, Santa Cruz, USA, 1:100), and ZP3 (rabbit polyclonal, Abcam, USA, 1:100) overnight at 4°C. Incubation was continued with FITC-conjugated donkey anti-rabbit IgG (Invitrogen, USA, 1:400) for ZP3, donkey anti-goat IgG (Invitrogen, USA, 1:400) for GDF9, donkey anti-mouse IgG (Invitrogen, USA, 1:400) for OCT4, and donkey anti-Rabbit IgG, Alexa Fluor 546 for VASA and DAZL for one hour at 37°C. Nuclei were stained with 1 µg/mL 4’,6-diamidino-2phenylindole (DAPI, Sigma, USA) for 5 minutes. For negative controls, the secondary antibodies were used alone. Images were taken with a fluorescence microscope (Eclipse 50i, Nikon, Japan).

## Statistical analysis 

All experiments were performed with three independent biological replicates, and the data were analyzed using one-way ANOVA (SPSS version 16.0), followed by Tukey’s test. Differences were considered significant at P<0.05.

## Results

**Fig.1:**
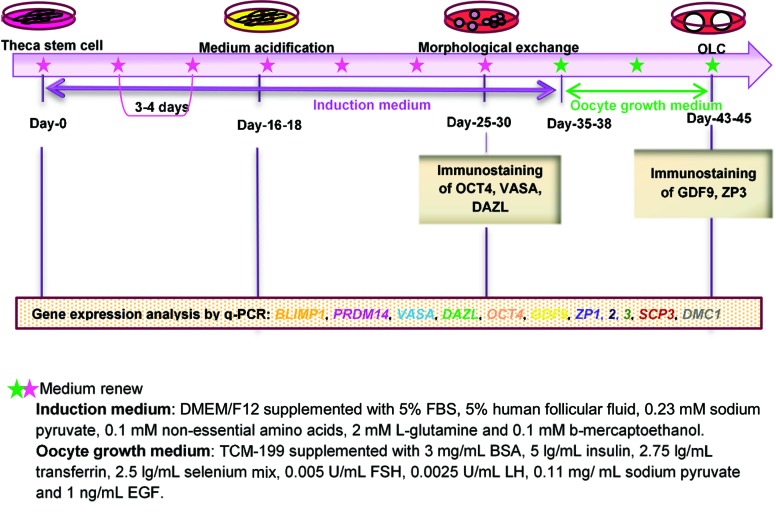
Schematic representation of hTSC differentiation into hOLCs. hTSCs; Human theca stem cells, hOLCs; Human oocyte like cells, DMEM/F12; Dulbecco’s Modified Eagle’s medium F12, FBS; Fetal bovine serum, BSA; Bovine serum albumin, FSH; Follicle-stimulating hormone, and LH; Luteinizing hormone.

**Fig.2:**
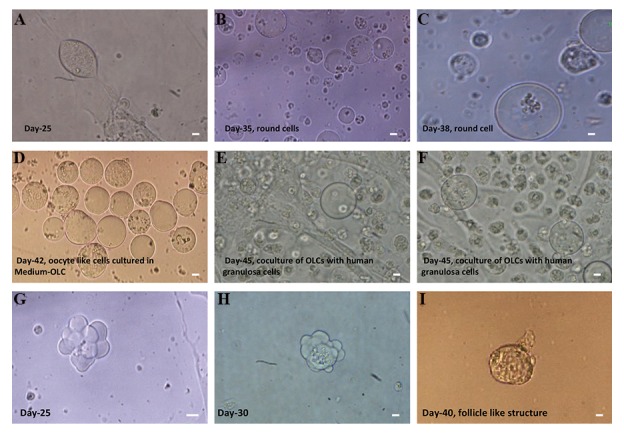
Differentiation of hTSCs into hOLCs in specific culture medium. A-D. Round cells 20-25 µm in diameter were observed at day 25 to approximately day 40 of differentiation, E, F. hOLCs could be further developed when cultured in OLC medium and co-cultured with human granulosa cells, and G-I. Differentiation of hTSCs into follicle-like structures. Cell aggregates, which resemble follicle-like structures, appeared at days 25-30 of differentiation cells (scale bars: 10 µm). hOLCs; Human oocyte like cells and hTSCs; Human theca stem cells.

**Fig.3:**
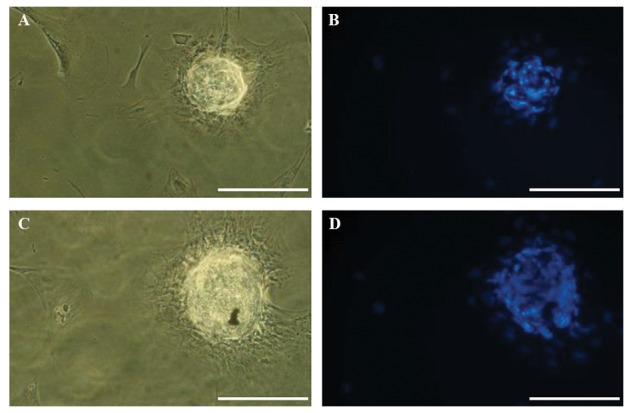
Differentiation of human theca stem cells (hTSCs) into colony-like structures. A, B. After 45 days of culture, the morphology of hTSCs was changed to a blastocyst-like structure, which was surrounded by cumulus-like cells, C, and D. Nuclear staining with DAPI is shown (scale bars: 200 µM).

As described in the methods and materials section, cells were cultured for 50 days in oocyte induction medium in order to differentiate hTSCs to hOLCs (Fig .1). After 16-18 days, the color of the medium indicated that it had become acidic. After 25 days, the cells started to differentiate into round-shaped cells (Fig .2). After 45-50 days, the morphology of hTSCs changed to colony-like structures, which showed a large number of nuclei similar to blastocysts, as visualized using DAPI (Fig .3). 

The morphology of the round cells seemed to be similar to that of oocytes. The average size and the number of round cells increased following induction (20-25 µm diameter). For further growth, the round cells were harvested and co-cultured with hGCs for one week, as described in the methods and materials section. During the co-culture period, the size of the round cells increased to 60 to70 µm. 

**Fig.4:**
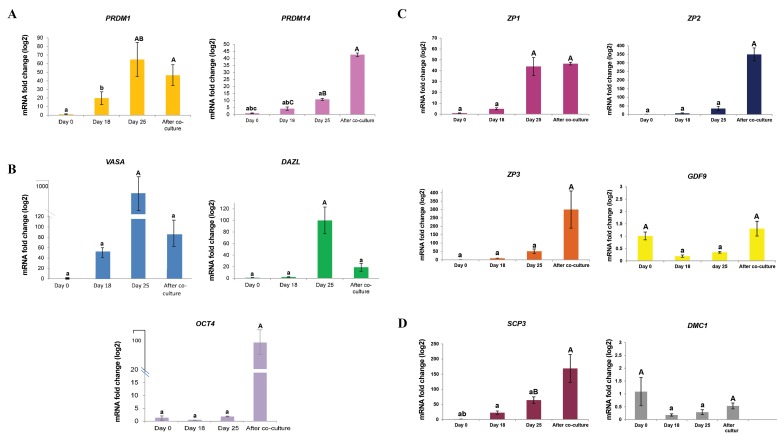
Expression of primordial germ cell, germ cell, oocyte and meiotic markers in human oocyte like cells. A. The detection of primordial germ cells (PRDM1, 14), B. Germ cells (VASA, DAZL, and OCT4), C. Oocytes (ZP1,^2^,^3^, and GDF9), and D. Meiotic markers (SCP3 and DMC1). ß-Actin was used as the internal control. The data were analyzed using ANOVA. Capital letters versus same small letters (A with a, B with b and C with c) indicated significantly different (P<0.05).

During differentiation of hOLCs, the expression of PGC and germ cell (*PRDM1, PRDM14, VASA, DAZL,* and *OCT4*), oocyte (*ZP1, 2, 3* and *GDF9*) and meiotic markers (*SCP3* and *DMC1*) was evaluated on days 0, 18, and 25 after monoculture and one week after co-culture with hGCs. Excluding *ZP2,3* and *SCP3* genes, the transcripts of all the markers were detected in the PGC-like cells (day 18) and germ-cell-like cells (day 25) and one week after co-culturing with hGCs (hOLCs), as well as in the undifferentiated cells (day 0) (Fig .4). However, the expression of genes in the differentiated cells compared with that in the undifferentiated ones depicted obvious dynamic alterations during hTSCs to hOLCs differentiation. 

On day 18 of differentiation induction, when the PGC-like cells appeared, the mRNA levels of *PRDM1* and *PRDM14* increased compared with day 0. *PRDM1* expression increased more than 3-fold on day 25 compared with day 18 (P<0.05). After one-week of co-culture of round cells with hGCs, *PRDM1* expression did not change (Fig .4A) compared with that on day 25. The expression of *PRDM14* gradually increased up to one week after co-culture with hGCs. *PRDM14* levels increased more than 4-fold and 8-fold, compared with those on day 18 and 25, respectively (P<0.05).

From day 18 to 25, the expression levels of *VASA* and *DAZL* genes were similar to those of *PRDM14* and PRDM1. The highest expression of these genes occurred on day 25 (P<0.05). The expression of *OCT4* increased on day 25 of differentiation and afterwards (Fig .4B). The *OCT4* expression suddenly increased one week after co- culture with hGCs. The expression of this gene was significantly increased compared with all previous steps (P<0.05).

From day 25 to one week after co-culture with hGCs, the expressions of *GDF9, ZP2,* and *ZP3* significantly increased compared to day 18 and day 25. The *ZP1* gene expression increased more than 8-fold on day 25 compared with day 18 (P<0.05) and a similar expression level was found during co-culture with hGCs (Fig .4C). 

**Fig.5:**
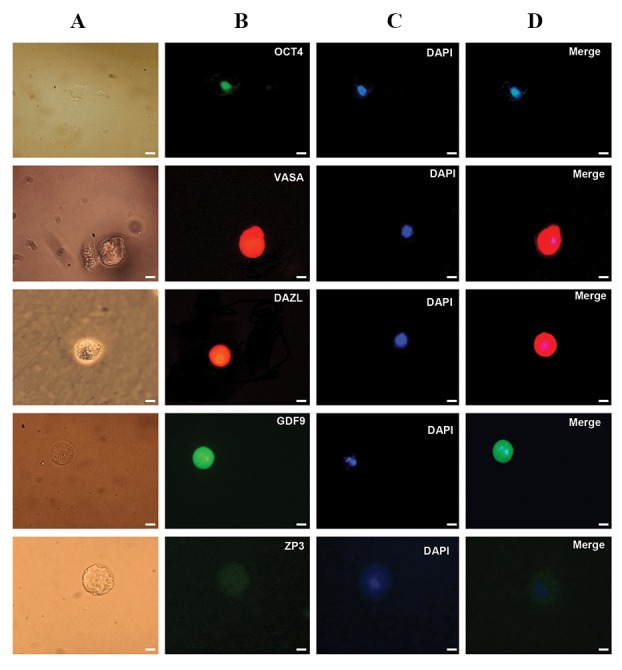
Immunofluorescence staining of germ cell markers (OCT4, VASA, and DAZL) in the round cells cultured in induction medium and oocytemarkers (GDF9 and ZP3) in hOLCs co-cultured with granulosa cells. A. Light microscopy images of the round cells on day 25-30, B. Immunofluorescence staining of OCT4, VASA, DAZL, GDF9, and ZP3 of the same cells, C. The round cells and human oocyte like cells (hOLCs) after DAPI staining, and D. Merged DAPI and primary antibody-secondary antibody-FITC staining of OCT4, VASA, DAZL, GDF9, and ZP3 in hOLCs. hOLCs (human oocyte such ascells (scale bars: 10 µm).

One week after co-culture with hGCs, the mRNA levels of *SCP3* and *DMC1* significantly increased compared with those on days 18 and 25 (P<0.05). However, the meiosis-specific marker *DMC1* was slightly expressed one week after co-culture with hGCs (Fig .4D). In addition, GDF9, OCT4, DAZL, VASA, and ZP3 proteins were present in hOLCs during same culture period (Fig .5).

## Discussion

Stem cell-derived artificial gametes have application in reproductive medicine and could benefit infertile couples. Despite findings concerning the differentiation of germ cells, more investigations need to be undertaken to elucidate the exact mechanism behind this process. Theca stem cells could be considered an alternative source for germ cells after gonadal insufficiency to induce cell differentiation (11, 14). 

We differentiated hTSCs into hOLCs in medium with 5% human follicular fluid. The culture medium became acidic after 18 days. This status may be due to changes in cell metabolism and initiation of cell differentiation into PGCs expressing *PRDM1, 14* and *VASA. PRDM1 *and *14* are key germ cell determinants for regulating PGC specification (15). Swartz (16) demonstrated that the metabolic activities of PGCs of chick embryo alter during migration. 

This can be justified in our study by changing the color of the culture medium for a short time. It seems that follicular fluid contains factors that are essential for differentiation of hTSCs to hOLCs. It has also been shown that follicular fluid because of estradiol hormone, might involve in differentiation of human OSE into OLCs (17). Approximately 7 days after the change in color of the culture medium, cells differentiated into round-shaped cells. Similarly, Dyce et al. (3) observed that some colony- like structures started to appear from stem cells of fetal porcine skin around day 20 of differentiation *in vitro*. At approximately day 30 of culture, some of the colonies detached from the growth surface and formed suspended cell aggregates. It has been showed that spherical cells appeared between days 14 and 30 after oogenic induction of porcine TSCs (11). In another study, Virant-Klun et al. (17) isolated 18 primitive oocyte-like cells that had differentiated from OSE in approximately 5 to 31 days. Yu et al. (18) also observed morphological changes of human amniotic fluid stem cells (hAFSCs) from a fibroblastoid type to a round shape after 5 days of incubation with differentiation medium.

Further, we used an extensive marker panel that represented genes expressed by germ cells and oocytes to evaluate the phenotype of the round cells. The round cells expressed *DAZL, VASA,* and *OCT4* genes, which have been shown to be required for germ cell formation. The presence of OCT4, VAZA, and DAZL proteins was confirmed by immunocytochemistry staining. Our data were in accordance with a previous report (11), which characterized porcine ovarian theca-derived multipotent cells and differentiated them to OLCs. However, the efficiency of this method of generating OLCs needs to be improved. 

In this study, the round cells were detached and transferred into oocyte growth medium containing gonadotropins and co-cultured with hGCs for 7-10 days. It has been reported that granulose cells induce the differentiation of ovarian stem cells into OLCs through cell-to-cell contact (19). Some of these cells grew larger and reached 60 to 70 µm in diameter. These cells were extracted to examine *GDF9, ZP1/2/3, SCP3,* and *DMC1* gene expression, as well as the presence of ZP3 and GDF9 proteins, to assess the similarity between the expressed markers and those of oocytes. 

The expression of *GDF9* (as a marker of normal folliculogenesis) (20) was evaluated at the mRNA and protein levels in the hOLCs. Our results showed that hOLCs also expressed oocyte-specific genes (*ZP1, 2, 3*) and ZP3 protein, although its expression intensity seemed much weaker than that of GDF9.

The meiosis-associated markers *DMC1* and *SCP3* were also expressed in the hOLCs, as previously explained. In agreement with our results, Lee et al. observed that OLC- derived TSCs express *c-MOS* and *SCP3* (11). In addition, Dyce et al. (3) showed that some of these large oocytelike cells express the marker *SCP3*. Furthermore, Yu et al. (18) demonstrated that *SCP3* was slightly expressed in embryo-like structures. Following this finding, the authors claimed that a small population of ES/iPS-derived OLCs but not somatic stem cell-derived OLCs were haploid. 

In summary, morphological similarities, gene expression, and protein presence implies that some of subpopulations of stem cells have ability to differentiate into hOLCs. Although the morphology of hTSCs-derived hOLCs was similar to that of the human oocyte but they were without zona pellucida and their size was smaller than the mature human oocyte (60-70 µm). These cells expressed both germ cell genes and some of the oocyte specific genes. Therefore, it appears that hOLCs are at the stage of the transition from germ cells to primary oocytes. Moreover, Linher et al. (21), demonstrated that somatic stem cells are able to differentiate into PGC-like cells in appropriate *in vitro* conditions, and then differentiate into germ cells expressing DAZL and oocyte-like cells.

## Conclusion

Taken together, we have demonstrated that hTSCs have the ability to differentiate into hOLCs. This introduced model paved the way for further *in vitro* studies of the exact mechanisms behind germ cell formation and differentiation. However, the functionality of hOLCs needs further elucidation. 
